# Evaluation of microvascular network with optical coherence tomography angiography (OCTA) in branch retinal vein occlusion (BRVO)

**DOI:** 10.1186/s12886-020-01405-0

**Published:** 2020-04-19

**Authors:** Lulu Chen, Mingzhen Yuan, Lu Sun, Yuelin Wang, Youxin Chen

**Affiliations:** Department of Ophthalmology, Peking Union Medical College Hospital, Chinese Academy of Medical Sciences, No.1 Shuaifuyuan Wangfujing Dongcheng District, Beijing, 100730 China

**Keywords:** Optical coherence tomography angiography, Retinal vasculature, Foveal avascular zone, Radial peripapillary capillary, Branch retinal vein occlusion

## Abstract

**Background:**

To evaluate changes of microvascular network of macular and peripapillary regions and to provide a quantitative measurement of foveal avascular zone (FAZ) in unilateral BRVO patients.

**Methods:**

Forty-seven unilateral BRVO patients and forty-seven normal controls were enrolled. A 3*3 mm scan centered on fovea followed by a 4.5*4.5 mm scan centered on optic nerve head (ONH) were obtained in BRVO eyes, fellow eyes and control eyes of each individual using OCTA (Optovue Inc., Fremont, CA, USA). Vessel density (VD) in superficial (SVC) and deep vascular complex (DVC) of macula and radial peripapillary capillary (RPC) were automatically calculated. Parameters of FAZ region including size, perimeter, acircularity index (AI) and foveal vessel density 300 (FD-300) were measured.

**Results:**

VDs of SCV and DVC were significantly lower, especially in affected regions, in BRVO eyes compared with fellow eyes (*P* < 0.05). BRVO affected eyes has larger FAZ size, FAZ perimeter, AI and lower FD-300 compared with fellow eyes (all *P* < 0.05). VD of SVC and FD-300 were lower in fellow eyes compared with normal control eyes (*P* < 0.05). The average vessel density in whole area and peripapillary area in BRVO eyes were significantly lower compared with fellow eyes (*P* < 0.05). VD of inside disc in fellow eyes was lower than normal eyes (*P* < 0.05).

**Conclusions:**

OCTA provided quantitative information of vascular changes in BRVO. FAZ in BRVO eyes showed significant morphological alterations and decreases of VD in surrounding area. Decreases of VD existed not only in SVC and DVC in macular region but also in RPCs in BRVO eyes. Unaffected eyes of unilateral BRVO showed vascular abnormalities in superficial retinal layer, peri-FAZ area and also peripapillary regions.

## Background

Retinal vein occlusion (RVO) is a retinal vascular diseases with high prevalence that can cause severe vison loss after diabetic retinopathy [1].

Systemic conditions, especially hypertension, hyperlipidemia and diabetes mellitus have been considered to be predisponding to RVO [2, 3]. Retinal vein occlusion can be categorized as central (CRVO), hemi-, and branch retinal vein occlusions (BRVO) based on the veins affected [4]. Fluorescein angiography(FA)is the gold standard for evaluating structural and functional status of retinal vasculature in retinal vascular diseases, especially in diabetic retinopathy and RVO. However, FA is an invasive procedure and it may lead to some side effects [5] such as allergic reaction and nausea. Details of retinal vessels may not be visualized because of dye leakage and pooling. It is also difficult to differentiate retinal vessels by layer, and deeper vasculature tissues are not well presented in FA images. Spectral-domain optical coherence tomography (SD OCT) is a noninvasive and efficient method to evaluate retinal and choroidal structures because of its ability to provide images with high resolution and has been widely used in scientific research and clinical settings. Optical coherence tomography angiography (OCTA), a more recent imaging facility, has enabled researchers to visualize microvascular in different retinal layers and choroid. It also provide us with quantitative information about perfusion status of macular and peripapillary regions. Using OCTA, microvascular changes such as microaneurysms, telangiectasia, retinal capillary nonperfusion and disruption of the foveal avascular zone have been found in RVO [6–8]. Decreased microvascular density in different vessel layers of macula and peripapillary region was also reported with quantitative analysis [8–11].

Recent research about FAZ mainly focused on the enlargement of its size and perimeter in diseased eyes, however, acircularity index (AI) has been proved to be a useful parameter depicting the asymmetry of FAZ area in retinal vascular diseases especially in diabetic retinopathy [12, 13]. Another quantitative parameter, foveal vessel density 300 (FD-300) is a new metric which evaluates vessel density of the area closely surrounding FAZ. To our knowledge, AI and FD-300 have not been used to evaluate FAZ asymmetry and perfusion states in BRVO patients.

The radial peripapillary capillary (RPC) layer is a delicately organized vasculature radiating from the optic nerve head (ONH) that lies most superficially. Cross sectional studies using OCTA has found a decreased RPC density in retinal vascular diseases, like branch and central retinal artery occlusions [14, 15]. Reports about RPC density from literature failed to differentiate RPCs and other large retinal vessels around the disc [16]. Microvascular changes in area surrounding the disc in patients with unilateral BRVO has not been analyzed thoroughly with quantitatively methods .

In this study, we measured AI and FD-300 as well as RPC density in BRVO eyes and fellow eyes in unilateral BRVO patients and we also compared these parameters between fellow eyes in BRVO patients and control eyes to present a thorough evaluation of microvascular changes in BRVO.

## Methods

### Patients

Forty-seven eyes of 47 patients with unilateral BRVO who were treated at the Department of Ophthalmology of Peking Union Medical College Hospital (PUMCH), Beijing, China between January 2018 and December 2018 were enrolled in this retrospective observational study. Treatment naïve patients and patients who have been treated with intravitreal medication were included. Patients with poor OCTA images quality (quality index below 5) because of poor eye fixation, media opacities were excluded. Subjects with retinal surgery history, pathologic myopia, ocular trauma, retinal artery occlusion, and other concomitant ocular diseases such as age-related macular degeneration, glaucoma and diabetic retinopathy were also excluded. The exclusion criteria also included eyes with CRVO or hemicentral retinal vein occlusion (HRVO).

One of the control groups consisted of the clinically unaffected fellow eyes of the enrolled subjects if the fellow eye was otherwise healthy without any known ocular diseases or ocular surgical operation. A normal anterior and posterior segment on examination of the eye and a normal intraocular pressure were required. Another control group was made up with 47 age-matched normal individuals who had no history of any ocular diseases or ocular surgical operation. The ophthalmic examination of the normal individuals was unremarkable. One eye of each normal individual was selected randomly as the control eye.

Information including age, sex, duration of disease and best corrected visual acuity (BCVA, logMAR) were obtained from the medical records. Each patients underwent a careful examination including slit lamp-assisted biomicroscopy, intraocular pressure, fluorescein angiography (FA), SD-OCT (Spectralis Heidelberg Engineering, Heidelberg, Germany), and OCTA using the AngioVue OCTA system version 2017.1 (Optovue Inc., Fremont, CA, USA).

### Macular microvascular OCTA imaging

Macular OCT angiograms images were captured using the AngioVue OCTA system version 2017.1 (Optovue Inc., Fremont, CA, USA) with the Angio Retina mode. A newly developed 3D projection artifact removal (PAR) algorithm was included in the program. In situ OCTA signal was differentiated from projection artifacts by the software based on the information from OCT and OCTA volume and removes the projection artifacts.

For each eye, a voxel image with a side of 3 mm centered on the fovea was chosen for analyze. The scanned vascularized tissue was automatically segmented into four enface slabs by the installed Angiovue software based on the default settings: the superficial vascular complex (SVC), the deep vascular complex (DVC), the outer retinal layer, and the choriocapillaris layer. Vascular tissue from the internal limiting membrane to 10 μm above the inner plexiform layer (IPL) consisted of the superficial capillary network. The deep capillary network was defined as vasculature from10 μm above the IPL to 10 μm below the outer plexiform layer (OPL), no overlap existed between the 2 slabs.

FAZ metrics including size, perimeter, foveal acircularity index(AI), and foveal vessel density 300(FD-300) were evaluated with the software(Table 3). AI is defined as the ratio between the measured perimeter and the perimeter of a circular area of the same size: the closer the shape is to the circle, the closer the value is to 1. FD-300 is the vessel density in a 300-mm wide area encompassing the FAZ, including both SVC and DVC. FAZ area is excluded in measurement of vessel density in this area, due to its high variability among different individuals. FD-300 is a useful parameter that gives us additional information about vasculature surrounding FAZ area and it has been used in the detection of early signs of diabetic retinopathy in previous studies [17].

### Radial peripapillary capillary measurement

A rectangle scan of 4.5*4.5 mm centered on ONH was obtained for each eye with AngioVue OCTA system using Angio-Disc mode. The software automatically fits a 2.0 mm diameter circle, centered on ONH, and defines a circle 2.0 mm wide that extends from the optic disc as the peripapillary region. The peripapillary area was divided into the following eight regions automatically based on Garway-Heath method [18]: nasal superior (NS), nasal inferior (NI), inferior nasal (IN), inferior temporal (IT). Temporal inferior (TI), temporal superior (TS), superior temporal (ST) and superior nasal (SN). Vessel densities of the whole image, inside disc and each sector of peripapillary area were generated by the software automatically.

The patients were divided into two sub groups based on the location (superior or inferior) of the affected vein and vessel density in each sector of the superficial and deep vascular plexus and RPC were compared in each group.

The automated layer segmentation and FAZ delineation in all scans were reviewed by two independent experts. In case of segmentation errors or FAZ delineation error, the examiners corrected errors manually until agreement achieved.

### Statistical analysis

Statistic analysis was performed by SPSS (SPSS for Mac, version 25.0; IBM/SPSS, Chicago, IL, USA). Continuous variables are sumerized as mean and SD. Paired *t* test was applied to compare the demographics and evaluate the difference in macular metrics, FAZ parameters and peripapillary vessel densities between BRVO eyes and the contralateral ones. Unpaired *t* test was applied between contralateral eyes and normal control ones. A two-tailed *P* value of < 0.05 as statistically significant.

## Results

### Patients’ demographic and clinical characteristics

Fifty-seven patients were enrolled primarily, while 10 patients were excluded. Out of the 10 excluded subjects, 7 patients were excluded because the quality of OCTA images were inadequate for analysis, 1 patient was excluded because of lack of FAZ zones in both eyes, 2 patients were excluded due to diabetic retinopathy. Forty-seven eyes of 47 patients (22 men) with a mean age of 55. 0 ± 11.0 years (median, 55.0 years; range, 25–82 years) were eventually included in this study. All patients had unilateral involvement. Thirty patients had right-eye involvement and 17 patients had left involvement. The superior temporal branch vein was occluded in 38 patients, the inferior temporal branch vein was occluded in 8 patients, and the superior nasal branch vein was occluded in 1 patient. The mean presenting BCVA was logMAR 0.440 ± 0.324. The mean duration of the symptoms of BRVO was 8.3 ± 14.7 months (0.5–46 months). The mean BCVA for the contralateral eyes was logMAR 0.096 ± 0.143 (Table 1). The foveal retinal thickness was significantly (*P* < 0.001) thicker in BRVO eyes than in the contralateral unaffected eyes. (Table 2). For the normal control group, the mean age was 53. 9 ± 13.03 years (median, 56.0 years; range, 27–73 years). The mean BCVA was logMAR 0.040 ± 0.116. The foveal retinal thickness was 248 ± 19 μm, not significantly differed from the contralateral unaffected eyes (*P* > 0.05) (Table 3).

**Table 1:** Demographics and clinical characteristics of patients with BRVO

Variables	Mean ± Standard Deviation
BRVO eyes	47
Age, y, mean ± SD	55. 0 ± 11.0 (range, 25–82)
Sex, male/female	22/25
Affected eye, OD/OS	30:17
BCVA of BRVO eyes	0.440 ± 0.324
BCVA of fellow eyes	0.096 ± 0.143
Symptom duration of BRVO	8.3 ± 14.7(range 0.5–46 months)
Superior/inferior, no	39/8

**Table 2:** Macular measurements in BRVO eyes and fellow eyes

Variables	Eyes with BRVO (*n* = 47)	Contralateral unaffected eyes (*n* = 47)	*P* value (paired *t* test)
BCVA	0.440 ± 0.324	0.096 ± 0.143	< 0.001
Foveal retinal thickness(μm)	321 ± 113	248 ± 20	< 0.001
Vascular density in SVC (%)			
Whole	40.4 ± 4.0	45.6 ± 3.5	< 0.001
Foveal	15.5 ± 5.0	15.5 ± 5.9	0.960
Temporal	41.3 ± 5.3	47.3 ± 3.5	< 0.001
Superior	42.2 ± 7.5	50.1 ± 3.9	< 0.001
Nasal	42.7 ± 6.0	47.5 ± 4.0	< 0.001
Inferior	44.6 ± 5.5	49.8 ± 4.0	< 0.001
Vascular density in DVC (%)			
Whole	42.9 ± 5.0	50.0 ± 3.0	< 0.001
Foveal	28.2 ± 9.3	29.8 ± 8.0	0.158
Temporal	44.0 ± 7.1	52.7 ± 3.0	< 0.001
Superior	41.1 ± 8.8	52.0 ± 3.6	< 0.001
Nasal	46.2 ± 6.4	52.8 ± 3.2	< 0.001
Inferior	46.0 ± 7.4	52.2 ± 3.8	< 0.001
FAZ(mm2)	0.394 ± 0.260	0.325 ± 0.136	0.046
FAZ perimeter(mm)	2.589 ± 1.108	2.255 ± 0.503	0.031
AI	1.23 ± 0.13	1.14 ± 0.04	< 0.001
FD-300(%)	45.66 ± 6.04	49.21 ± 4.46	< 0.001

**Table 3:** Macular measurements in contralateral eyes and normal control eyes

Variables	Contralateral unaffected eyes (*n* = 47)	Normal control eyes (*n* = 47)	*P* value (unpaired *t* test)
BCVA	0.096 ± 0.143	0.040 ± 0.116	0.056
Foveal retinal thickness(μm)	248 ± 20	248 ± 19	0.954
Vascular density in SVC (%)			
Whole	45.6 ± 3.5	47.2 ± 2.6	0.015
Foveal	15.5 ± 5.9	15.6 ± 6.6	0.963
Temporal	47.3 ± 3.5	48.8 ± 2.9	0.022
Superior	50.1 ± 3.9	51.5 ± 2.8	0.047
Nasal	47.5 ± 4.0	48.7 ± 5.3	0.204
Inferior	49.8 ± 4.0	51.1 ± 3.2	0.094
Vascular density in DVC (%)			
Whole	50.0 ± 3.0	50.5 ± 3.3	0.451
Foveal	29.8 ± 8.0	28.7 ± 5.5	0.803
Temporal	52.7 ± 3.0	53.7 ± 3.2	0.141
Superior	52.0 ± 3.6	53.0 ± 3.2	0.142
Nasal	52.8 ± 3.2	53.9 ± 2.9	0.090
Inferior	52.2 ± 3.8	52.9 ± 3.9	0.340
FAZ(mm2)	0.325 ± 0.136	0.352 ± 0.090	0.270
FAZ perimeter(mm)	2.255 ± 0.503	2.345 ± 0.348	0.316
AI	1.14 ± 0.04	1.14 ± 0.04	0.686
FD-300(%)	49.21 ± 4.46	51.39 ± 3.56	0.010

### Macular vessel density

Lower vessel density was detected in eyes affected by BRVO in both the SVC (*P* < 0.05) and DVC (*P* < 0.05) compared with the fellow eyes in OCTA images. In the BRVO group, the FAZ is more irregular in shape compared with that in the fellow eyes. The size (*P* < 0.05) and perimeter of FAZ (*P* < 0.05) were significantly larger in BRVO eyes than fellow eyes. The AI was higher (*P* < 0.05) and foveal vessel density 300 was lower (*P* < 0.05) in BRVO eyes compared with the fellow eyes (Table 2). The contralateral eyes showed lower SVC in whole area, especially in temporal area and superior area of macular region (*P* < 0.05), compared with normal control eyes. FD-300 was significantly lower in contralateral unaffected eyes than in normal control eyes (*P* < 0.05) (Table 3).

**Fig. 1:**
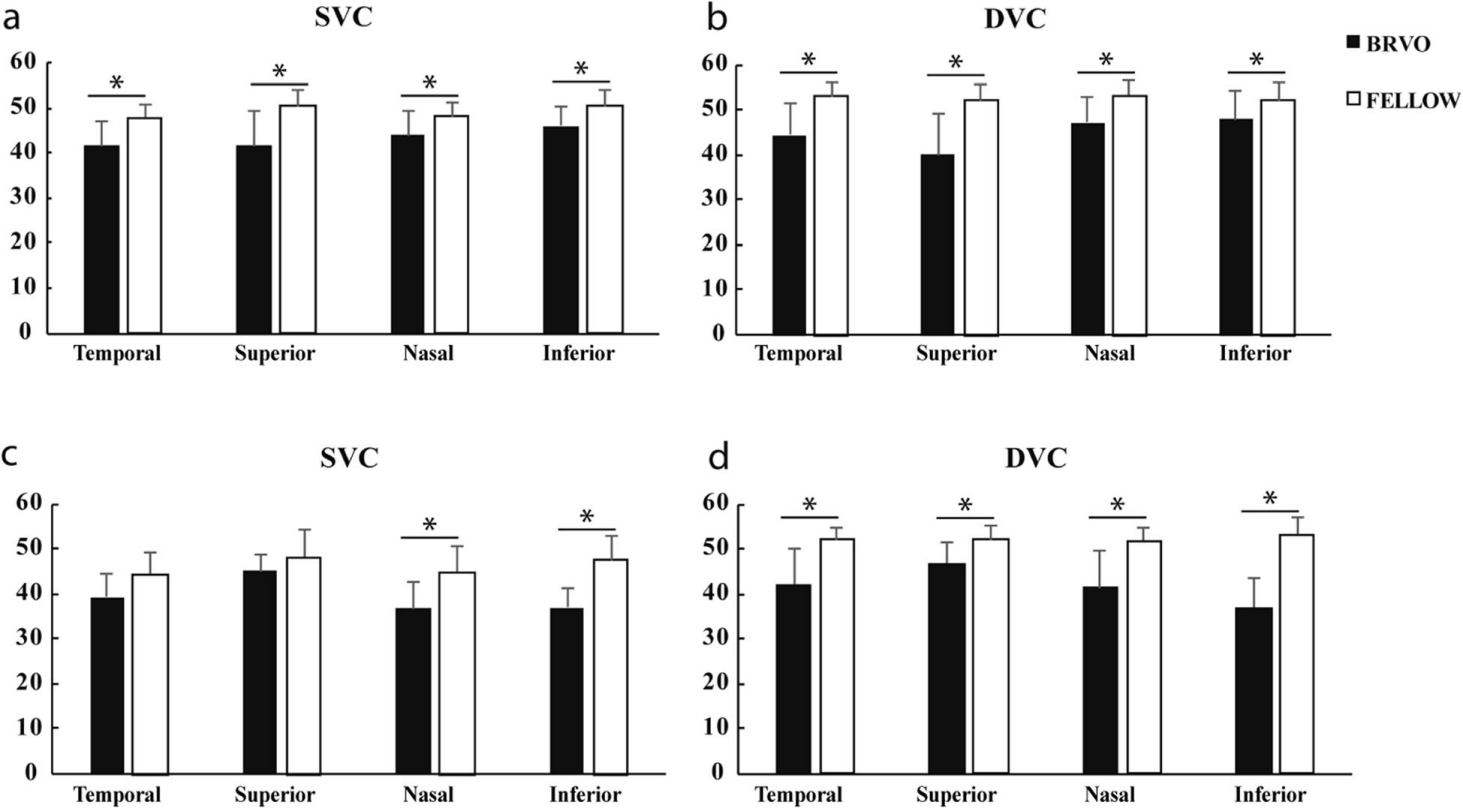
Vessel density of SVC and DVC (**a**-**b**) in macular region of superior vein occlusion group and (**c**-**d**) inferior vein occlusion group. *, *P* < 0.05; SVC, superficial vascular complex; DVC, deep vascular complex

In sub group analysis, we found vessel density was significantly lower in all sectors (*P* < 0.05) except foveal region in SVC and DVC in eyes with superior vein occlusion. Vessel density was significantly lower in inferior (*P* < 0.05) and nasal (*P* < 0.05) sectors in SVC and in all sectors except foveal region in DVC in eyes with inferior vein occlusion (Fig. 1).

### Peripapillary vessel density

The average vessel density of the whole, inside disc and peripapillary region were 47.7 ± 4.0, 46.8 ± 6.2, 49.9 ± 4.2 in BRVO eyes, and 49.9 ± 2.3, 47.7 ± 5.6, 52.7 ± 3.0 in contralateral eyes. BRVO eyes has lower vessel density in whole area and peripapillary area compared with contralateral eyes (*P*<0.05) (Table 4).

**Table 4:** Peripapillary vessel density in BRVO eyes and fellow eyes

Variables	Eyes with BRVO (*n* = 47)	Contralateral unaffected eyes (*n* = 47)	*P* value
Whole(%)	44.7 ± 4.0	49.9 ± 2.3	< 0.001
Inside disc(%)	46.8 ± 6.2	47.6 ± 5.4	0.390
Peripapillary(%)	47.8 ± 5.6	52.8 ± 3.1	< 0.001
Nasal superior(%)	44.1 ± 5.6	48.9 ± 4.7	0.159
Nasal inferior(%)	47.2 ± 5.8	47.9 ± 4.8	0.410
Inferior nasal(%)	49.4 ± 9.1	52.5 ± 4.2	0.035
Inferior temporal(%)	52.9 ± 9.3	58.4 ± 4.5	< 0.001
Temporal inferior(%)	52.4 ± 6.4	53.3 ± 5.0	0.337
Temporal superior(%)	54.4 ± 4.2	56.7 ± 3.7	0.005
Superior temporal(%)	49.0 ± 7.9	56.6 ± 4.1	< 0.001
Superior nasal(%)	47.4 ± 7.4	51.3 ± 4.5	< 0.001

The average vessel density of the whole, inside disc and peripapillary region were 50.7 ± 1.6, 50.7 ± 5.3, 53.3 ± 2.2 in normal control eyes. The contralateral unaffected eyes has lower VD in whole area and inside disc area of the peripapillary region (*P*<0.05) (Table 5) compared with normal eyes.

**Table 5:** Peripapillary vessel density in contralateral unaffected eyes and normal control eyes

Variables	Contralateral unaffected eyes (*n* = 47)	Normal control eyes (*n* = 47)	*P* value
Whole(%)	49.9 ± 2.3	50.7 ± 1.6	0.040
Inside disc(%)	47.6 ± 5.4	50.7 ± 5.3	0.007
Peripapillary(%)	52.8 ± 3.1	53.2 ± 2.2	0.467
Nasal superior(%)	48.9 ± 4.7	49.1 ± 4.0	0.798
Nasal inferior(%)	47.9 ± 4.8	48.7 ± 4.1	0.394
Inferior nasal(%)	52.5 ± 4.2	53.0 ± 3.6	0.588
Inferior temporal(%)	58.4 ± 4.5	58.4 ± 3.5	0.953
Temporal inferior(%)	53.3 ± 5.0	53.7 ± 3.3	0.642
Temporal superior(%)	56.7 ± 3.7	57.6 ± 2.6	0.174
Superior temporal(%)	56.6 ± 4.1	56.9 ± 3.1	0.719
Superior nasal(%)	51.3 ± 4.5	51.2 ± 4.2	0.855

**Fig. 2:**
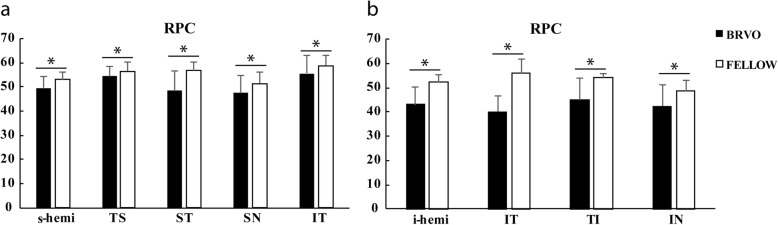
Vessel density of RPC in **a** superior vein occlusion group and **b** inferior vein occlusion group. RPC, radial peripapillary capillary; s-hemi, superior -hemi; i-hemi, inferior-hemi TS, temporal superior; ST, superior temporal; SN, superior nasal; IT, inferior temporal; TI, temporal inferior; IN, inferior nasal; *, *P* < 0.05

In eyes with superior vein occlusion, vessel density in inferior temporal, temporal superior, superior temporal and superior nasal sectors were significantly lower than contralateral eyes (*P*<0.05). In eyes with inferior vein occlusion, vessel density in inferior nasal, inferior temporal and temporal inferior sectors were significantly lower than contralateral eyes (*P*<0.05) (Fig. 2).

## Discussion

In this study, we quantified vessel density in macular region using OCTA. Our study revealed that vessel density in BRVO eyes was significantly lower in affected and adjacent sectors in superficial retinal layer and in all regions but the foveal region in deep retinal layer compared with contralateral unaffected eyes.

The decrease of vessel density in macular region was in accordance to previous studies that applied OCTA to measure vessel densities in retinal vein occlusion patients [7, 19–22]. In a previous BRVO study by Samara’s group, vessel densities in macular region were measured by sector and researchers found vessel density in affected sector of BRVO eyes was lower than fellow eyes in both superficial and deep network, while vessel density in the unaffected sector of BRVO eyes was also lower than fellow eye in deep network [23]. What’s more, affected sector was defined as superior or inferior quadrant of a circular grid in Samara’s study, so they only compared superior and inferior sectors in their research. Changes in nasal and temporal regions were not discussed in their work. In the study of Coscas et al. they found that nonperfusion areas were more common in deep capillary plexus [7], suggesting that DCPs were more vulnerable to ischemic attack. Freund et al. discovered that collateral vessels mainly existed in deep vascular complex, supporting a continuous arrangement of superficial and deep vascular complexes and venous drainage through the deep vascular complex [24]. Agreed with previous study, our result suggested DCP was more vulnerable to ischemic changes and BRVO may cause a more extensive vascular alteration.

Recent studies found enlarged FAZ in superficial and deep retinal layer in BRVO eyes [23, 25, 26], which was negatively correlated with visual acuity [23, 25, 27]. In our study, FAZ size and perimeter were larger in BRVO eyes compared with fellow eyes, which agreed with previous reports. Besides, we also found a larger AI and lower FD-300 in BRVO affected eyes in comparison to fellow eyes. Wons et al. measured the angle between the papillomacular plane and the maximum FAZ diameter in RVO eyes and contralateral unaffected eyes and found a significant difference between the two groups. According to their research, a distorted FAZ and an unorganized capillary structure would be a marker in RVO affected eyes [28]. Previous studies has suggested that FAZ size may vary considerably in normal individual and causing a significant overlap among healthy and diseased individuals [29, 30], making it difficult to recognize difference between study groups. Recently, a new parameter AI was described by Tam et al. to quantify the irregularity of FAZ [13], which has been proved to be a useful parameter evaluating the asymmetry of the FAZ, providing information about the ischemic status of diseased eyes [12]. Our result agreed with Won’s study and showed that BRVO affected eyes are more irregular in shape compared with fellow eyes. This was the first study a, to describe the asymmetry of FAZ in BRVO eyes with AI. Decrease in FD-300 reflects vascular drop out around foveal region and has been proved to be a useful parameter in diabetic retinopathy [17]. No significant differences were detected in foveal vessel density between superficial and deep vessel complex in our study. This could be the caused by the scarcity of vessels in FAZ region. However, when the quantified area skipped FAZ and extend to 300 μm area surrounding FAZ, vascular drop out became obvious. A lower vessel density of SVC in contralateral unaffected eyes compared with normal control eyes was another discovery in our study, which agreed with Wang’s research [19]. This may suggest that BRVO may be the result of systemic changes in both eyes and vascular drop out may have happened prior to BRVO event. Interestingly, decrease of VD was only found in SVC in contralateral eyes, which also agrees with Wang’s result. This may indicate that the underlying mechanism of vascular drop out in contralateral eyes was something other than the blockage of vessels in BRVO eyes. Besides, we also found that FD-300 in contralateral eyes was also lower compared with normal control eyes, while no difference was found regarding AI, FAZ size, and FAZ perimeter between the two groups. This may indicate that vascular drop out around FAZ is sensitive in the contralateral eyes.

Radial peripapillary capillary dropout was another important discovery in our study. Wang et al. measured flow velocity of veins merging from optic disc and found a slower flow velocity in the occluded hemisphere [31]. Recent studies found a reduction of peripapillary choroidal thickness in BRVO affected and fellow eyes in unilateral BRVO subjects, suggesting BRVO may be associated with a hypoxic attack to the peripapillary choroid [11, 32]. Shin et al. discovered that the peripapillary vessel density and perfusion density were lower in the fellow eyes of unilateral BRVO patients [16], indicating RVO may cause structural abnormality even in fellow unaffected eyes. However, Wang’s study only measured large vessels around optic disc while Shin’s research measured both large vessels and radial peripapillary capillaries. In our study, we measured radial peripapillary capillaries around optic nerve head with the installed software and found a significant decrease of peripapillary radial peripapillary capillary density in the affected sectors of BRVO eyes. Similar to Shin’s research, we also found vascular drop out of peripapillary vessels in contralateral unaffected eyes. However, vascular drop out stands out in the inside disc region, which disagrees with Shin’s study. Since we use different apparatus and the area we measured was different, the result could be influenced by these factors. Further study is in need to reveal the most vulnerable area of vascular drop out in peripapillary regions. As far as I know, this is the first report to quantify radial peripapillary capillary change in BRVO affected eyes.

In normal human eyes, blood supply to ONH is relying on posterior ciliary artery and central retinal artery, which also plays a role in blood supply of superficial RNFL layer of ONH. RPCs are straight, oriented superficial vessels that are paralleled to each other and arched to nourish RNFL around the ONH [33–35]. In the past decades, fluorescein angiography (FA) was used in the evaluation of ONH perfusion. However, FA requires dye injection and it is difficult to observe RPCs with this traditional method. OCTA shows the advantage for evaluating ONH perfusion because of its non-invasive nature and high resolution. RPCs can be easily visualized and vascular perfusion status can be quantified within minutes with OCTA. RPC drop out has been reported in glaucoma [36] and diabetes mellitus [37] patients, and has been considered as a useful parameter evaluating vascular dysfunction. Current research has found that mean RPC density was correlated with mean RNFL thickness [34]. Decreasing of RNFL thickness was found with OCT in RVO eyes [32, 38, 39], suggesting retinal nerve fiber atrophy may be the result of RVO as the disease progressed. In our study, PRC was lower in BRVO eyes in comparison to fellow eyes, especially in the affected sectors. However, no significant thinning of RNFL was observed in either the affected sector or the unaffected sector (data not shown), which disagreed with previous studies. This could be explained by the discrepancy of patients included in our research since we included both treatment naïve patients and those who has been treated. ONH swelling is usually observed in treatment naïve patients at the early stage of BRVO and this can influence the average RNFL thickness. However, the underlying swelling of RNFL may have little impact on RPC and the decrease of RPC density was prominent in our research.

Some limitations remains in this research. First, the study is a retrospective study and lacks longitudinal data. A longitudinal study is necessary to elucidate vascular changes with treatments. Besides, there is likely a selection bias because the sample size was limited. In addition, we did not measure vessel density of choriocapillaris in macular and ONH region. Artifacts such as projection and shadow may influence the accuracy of the measurement of choriocapillaris [40]. We scanned a 3*3mm^2^ area of the foveal and 4.5*4.5mm^2^ area of the ONH and these regions were relatively small. A larger scanning region with high accuracy may bring out more information of perfusion status. Further studies are needed to provide a thorough status of perfusion and vascular changes in a larger scale in BRVO eyes with the updated OCTA techniques.

## Conclusions

In conclusion, our study demonstrated OCTA with upgraded software enhanced with 3D PAR provides high-resolution images and quantitative information of microvascular parameters of not only macular, but also peripapillary vascular in BRVO eyes. Vascular density of SVC and DVC in macular region was much lower in BRVO eyes compared with fellow eyes. SVC was significantly lower in affected sectors while all sectors in DVC were decreased in affected eyes. In addition to enlarged FAZ area and perimeter, larger AI and lower FD-300 are prominent in BRVO eyes and can be used to evaluate the asymmetry and ischemic status of foveal region. More importantly, lower RPC density was another feature of BRVO affected eyes and RPC density can be a useful parameter to evaluated perfusion status of ONH in BRVO. Unaffected eyes of unilateral BRVO showed vascular abnormalities in superficial retinal layer, peri-FAZ area and also peripapillary regions.

## Data Availability

The datasets presented in this study is available from the corresponding author upon request.

## Abbreviations

RVO: Retinal vein occlusion
FAZ: Foveal avascular zone
ONH: Optic nerve head
VD: Vessel density
SVC: Superficial vascular complex
DVC: Deep vascular complex
RPC: Radial peripapillary capillary
AI: Acircularity index
FD-300: Foveal vessel density 300
CRVO: Central retinal vein occlusion
SD-OCT: Spectral-domain optical coherence tomography
HRVO: Hemicentral retinal vein occlusion
BCVA: Best corrected visual acuity
FA: Fluorescein angiography
IPL: Inner plexiform layer
OPL: Outer plexiform layer
NS: Nasal superior
NI: Nasal inferior
IN: Inferior nasal
IT: Inferior temporal
TI: Temporal inferior
TS: Temporal superior
ST: Superior temporal
SN: Superior nasal
